# Rapidly adapting primary care sentinel surveillance across seven countries in Europe for COVID-19 in the first half of 2020: strengths, challenges, and lessons learned

**DOI:** 10.2807/1560-7917.ES.2022.27.26.2100864

**Published:** 2022-06-30

**Authors:** Jayshree Bagaria, Tessa Jansen, Diogo FP Marques, Mariette Hooiveld, Jim McMenamin, Simon de Lusignan, Ana-Maria Vilcu, Adam Meijer, Ana-Paula Rodrigues, Mia Brytting, Clara Mazagatos, Jade Cogdale, Sylvie van der Werf, Frederika Dijkstra, Raquel Guiomar, Theresa Enkirch, Marta Valenciano, I-MOVE-COVID-19 study team, I-MOVE-COVID-19 study team, Esther Kissling, Lisa Domegan, Joan O’Donnell, Josephine Murray, Virginia Sandonis Martín, Iván Martínez-Baz, Ausenda Machado, Itziar Casado, Sylvie Behillil, Amparo Larrauri, Ruby Tsang, Marit de Lange, Maximilian Riess, Jesús Castilla, Mark Hamilton, Alessandra Falchi, Francisco Pozo, Linda Dunford, Cristina Burgui, Debbie Sigerson, Thierry Blanchon, Eva María Martínez Ochoa, Jeff Connell, Joanna Ellis, Rianne van Gageldonk-Lafeber, Irina Kislaya, Angela MC Rose, Jamie Lopez Bernal, Nick Andrews, Inmaculada Casas Flecha, Janine Thoulass, Baltazar Nunes, Verónica Gomez, Rita Sa Machado, Vincent Enouf, Pedro Licinio Pinto Leite, Anna Molesworth, Adele McKenna, Janine Thoulass

**Affiliations:** 1 Public Health Scotland, Glasgow, Scotland; 2 Nivel, Utrecht, The Netherlands; 3 Epiconcept, Paris, France; 4 Nuffield Department of Primary Care Health Sciences, University of Oxford, Oxford, United Kingdom; 5 Royal College of General Practitioners Research and Surveillance Centre, London, United Kingdom; 6 INSERM, Sorbonne Université, Institut Pierre Louis d'épidémiologie et de Santé Publique (IPLESP UMRS 1136), Paris, France; 7 National Institute for Public Health and the Environment, Bilthoven, the Netherlands; 8 Instituto Nacional de Saúde Dr. Ricardo Jorge, Lisbon, Portugal; 9 The Public Health Agency of Sweden, Stockholm, Sweden; 10 National Centre for Epidemiology, Institute of Health Carlos III, Madrid, Spain; 11 Health Security Agency, London, United Kingdom; 12 Institut Pasteur, Université Paris Cité, CNRS UMR 3569, Molecular Genetics of RNA viruses unit, National Reference Center for Respiratory Viruses, Paris, France; Unité de Génétique Moléculaire des Virus à ARN, UMR 3569 CNRS, Université Paris Diderot SPC, Institut Pasteur, Paris, France; 13 The members of the I-MOVE-COVID-19 primary care study team are listed under Collaborators

**Keywords:** Sentinel surveillance, primary care, SARS-CoV-2, Influenza-Like Illness (ILI), Europe, COVID-19

## Abstract

As the COVID-19 pandemic began in early 2020, primary care influenza sentinel surveillance networks within the Influenza - Monitoring Vaccine Effectiveness in Europe (I-MOVE) consortium rapidly adapted to COVID-19 surveillance. This study maps system adaptations and lessons learned about aligning influenza and COVID-19 surveillance following ECDC / WHO/Europe recommendations and preparing for other diseases possibly emerging in the future. Using a qualitative approach, we describe the adaptations of seven sentinel sites in five European Union countries and the United Kingdom during the first pandemic phase (March–September 2020). Adaptations to sentinel systems were substantial (2/7 sites), moderate (2/7) or minor (3/7 sites). Most adaptations encompassed patient referral and sample collection pathways, laboratory testing and data collection. Strengths included established networks of primary care providers, highly qualified testing laboratories and stakeholder commitments. One challenge was the decreasing number of samples due to altered patient pathways. Lessons learned included flexibility establishing new routines and new laboratory testing. To enable simultaneous sentinel surveillance of influenza and COVID-19, experiences of the sentinel sites and testing infrastructure should be considered. The contradicting aims of rapid case finding and contact tracing, which are needed for control during a pandemic and regular surveillance, should be carefully balanced.

## Introduction

As the coronavirus disease (COVID-19) pandemic is not over and the severe acute respiratory syndrome coronavirus (SARS-CoV-2) will likely become endemic [1], it is important to reflect on systems to detect and monitor the spread and evolution of the virus. Moreover, careful consideration is needed to prepare sentinel surveillance systems for the future, when testing for SARS-CoV-2, influenza viruses and possibly other viruses with public health impact, such as respiratory syncytial virus (RSV), needs to be integrated.

The first cases of COVID-19 in Europe were identified on 24 January 2020 in France, and hereafter, uncontrolled community transmission was detected in the United Kingdom (UK) and Italy in February, and in Spain in early March. Most European countries have shown that consistent application of societal and public health measures can slow person-to-person spread of SARS-CoV-2 [2]. These measures included for instance isolation, contact tracing, quarantine of contacts and detection of active cases through testing. In addition, public health interventions included physical distancing, hand hygiene and widespread use of face masks in public [2]. Whereas the surge of outbreaks and the timing of measures taken differed across countries, most countries at some point imposed lockdowns and curfews, effectively shutting down non-essential economic activity to minimise in-person interactions to contain the virus [3]. However, many countries experienced a surge in cases when measures were relaxed [3].

In March 2020, the World Health Organization (WHO) recommended that in addition to outbreak investigation and management, countries should set up and maintain enhanced influenza surveillance activities. Rather than setting up new systems, the European Centre for Disease Prevention and Control (ECDC) and WHO recommended that, when possible, countries should adapt existing respiratory disease surveillance systems to monitor the spread of COVID-19, such as hospital-based severe acute respiratory infection (SARI), primary care acute respiratory infection (ARI) and influenza-like illness (ILI) [4,5]. These surveillance systems should be used to detect and monitor community transmission of SARS-CoV-2 according to four transmission scenarios – no cases, sporadic cases, clusters of cases, and community transmission [6] with the aims to (i) monitor geographical spread, severity, and intensity of transmission; (ii) collect genomic information to be considered in the development of drugs and vaccines; (iii) collect data on risk factors for disease to enable targeted prevention; (iv) monitor the impact on health systems; and (v) monitor the impact of mitigation measures [4].

In Europe, influenza surveillance is performed jointly by ECDC and the WHO Regional Office for Europe (WHO/EURO). The data from the countries’ weekly reports are summarised in the Flu News Europe, a weekly bulletin produced by the two organisations [7]. Data related to SARS-CoV-2 detection and COVID-19 epidemiology were rapidly added to the weekly reports. In addition, 15 countries across Europe participate in the Influenza-Monitoring Vaccine Effectiveness in Europe (I-MOVE) network to share information and estimate influenza vaccine effectiveness across influenza seasons, including both primary and secondary care [8]. Established in 2007, the I-MOVE network includes primary care, hospital, and laboratory surveillance networks that measure influenza vaccine effectiveness. The network has vast experience in multicentre studies. Sentinel primary care practitioners collect specimens from a sample of patients who present with ILI or ARI within 8 days of symptom onset. These specimens are tested at regional reference laboratories. Participating countries/regions adapt the generic protocol to their specific situation [8]. In response to the COVID-19 pandemic, this network was expanded to include COVID-19 in the I-MOVE-COVID-19 Consortium. The expansion was designed to strengthen surveillance systems in the participating countries so that European countries detected and responded to COVID-19 cases as rapidly as possible [9].

The I-MOVE-COVID-19 primary care network aims to share information and to conduct studies on sentinel surveillance for COVID-19 to better understand the virus and its spread [7]. The network comprises six sentinel sites in European Union (EU) countries (France, Ireland, the Netherlands, Portugal, Spain and Sweden) and two sites in the UK (England and Scotland) (see Supplementary Table S1 for a description of the network).

Here we present the experiences of these I-MOVE-COVID-19 network countries in adapting their primary care influenza sentinel surveillance systems for COVID-19 surveillance. Our objectives are to map the adaptations to the surveillance systems during the first pandemic phase (March–September 2020) and to identify the strengths, challenges and lessons learned from the perspective of seven participating sentinel sites. Our ultimate aim is to use this information to prepare future integration of COVID-19 and influenza sentinel surveillance and strengthen preparedness. With new SARS-CoV-2 variants emerging worldwide and the relaxation of social and testing measures, the importance of virus detection and characterisation through primary care sentinel surveillance is warranted even more [10], while ‘the threat of influenza epidemic and pandemics persist’ [11].

## Methods

Data on country surveillance systems were collected between July and September 2020 using a qualitative mixed methods approach. One participating site, Ireland, was in the process of adapting the Irish sentinel surveillance system at the time of data collection. Data collection and analysis were performed in five steps of which steps 4 and 5 were developed in response to information collected during steps 1–3 (Figure).

**Figure:**
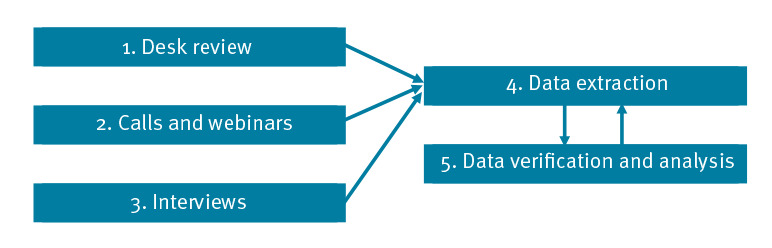
Steps in information collection on adaptations to surveillance systems in six European countries^a^ during the first pandemic phase, March–September 2020

^a^ France, the Netherlands, Portugal, Spain, Sweden and two sites in the United Kingdom (England and Scotland).

### Step 1. Desk review

The desk review step included ECDC, WHO COVID-19 publications and I-MOVE-COVID-19 network country policy documents on influenza and COVID-19 surveillance, including testing policies and protocols. Documents from ECDC and WHO/Europe were identified by reviewing all technical guidance found on the organisations’ website and from references presented during WHO webinars [5,7,11-15]. During the weekly ECDC/WHO teleconferences, there were country presentations and they provided references. Guidelines and reports from ECDC and WHO were announced on their websites and also during these teleconferences. Partners involved in this ‘lessons learned’ paper provided additional references. Country profiles on influenza sentinel surveillance were sourced from the WHO/Europe website [7]. Additional published and unpublished information was provided by the I-MOVE-COVID-19 network country focal points [16,17].

### Step 2. Calls and webinars

I-MOVE-COVID-19 project teleconferences and informal meetings or communications allowed participants to share their experiences adapting primary care sentinel surveillance systems. The participants took part in weekly joint ECDC and WHO/Europe teleconferences on COVID-19, during which country experiences were presented, as well as results of the ECDC sentinel surveillance survey in April–June 2020.

### Step 3. Interviews

Semi-structured interviews were conducted with I-MOVE-COVID-19 consortium primary care focal points and leads from England, Scotland and Sweden and the primary care lead and I-MOVE-COVID-19 network secretariat from the Netherlands in September 2020. At the time of the interviews, the other study sites did not have their COVID-19 sentinel surveillance systems operational. The interview template can be found in Supplement S3.

### Step 4. Data extraction

Data from steps 1–3 were extracted and used to develop an Excel template. The template was cross-checked and shared with focal points from participating I-MOVE-COVID-19 network countries for further input. Using the template, we synthesised self-reported data on influenza sentinel surveillance structures from the survey from countries across three time periods: (i) pre-pandemic (before March 2020), (ii) peak of COVID-19 first wave (March–May 2020) and (iii) after the first wave peak (June–September 2020). The survey questions were divided into the sections (i) Sentinel surveillance system implementation and (ii) Reflections on strengths, challenges and lessons, each containing three items (Box).

Box **Data collected in survey on adaptations to surveillance systems in six European countries^a^ during the first pandemic phase, March–September 2020**

**Section 1. Sentinel surveillance system implementation**

• Swab collection: initiating or requesting site; place of and person who swabs; sampling criteria; and structure of surveillance, being population-based or sentinel.

• Data collection: format; person responsible; means of collection; and validation.

• Testing process: transport and logistics; location; tests conducted; results processing.

**Section 2. Reflections on strengths, challenges and lessons**

• Barriers at each stage;

• Enablers at each stage;

• Lessons learned.

^a^ France, the Netherlands, Portugal, Spain, Sweden and two sites in the United Kingdom (England and Scotland).

When additional information or clarification was needed, telephone or Zoom interviews were arranged with country focal points.

### Step 5. Data verification and analysis

Data were cross-checked by colleagues in the I-MOVE-COVID-19 Consortium. Countries were graded according to whether changes were made to the three items noted under ‘Sentinel surveillance system implementation’. Countries were classified as having made minimal or no changes if changes were only made to one of the three items, moderate if changes were made to two of the items or major if changes were made to all three items. Findings were shared and cross-checked with participating countries and the I-MOVE-COVID-19 secretariat.

## Results

Seven of eight sentinel sites responded to the call for input. Adaptions to the sentinel surveillance systems and strengths and challenges, and lessons learned are summarised by sentinel site in Supplementary Table S2. The following is a description of the adaptations of the sentinel surveillance systems, the strengths and challenges of the systems and the lessons learned in adapting the systems.

### Adaptations to sentinel surveillance system

The adaptations to the sentinel surveillance systems mainly addressed data collection, patient pathways, sampling criteria for swabbing and decentralisation of laboratory capacity. The Table shows the configurations of the sentinel surveillance that were adapted (and to what degree) for each of the study sites. To consider the impact of the major adaptations to patient pathways that were issued in almost half of the countries, we disentangled the first item in Section 1 (swab collection) into two items in the Table: patient pathway and sampling criteria.

**Table:** Most commonly adapted aspects to sentinel surveillance systems by country and degree of change, during the first pandemic phase, March–September 2020

Country	Degree of changes			
	Swab collection		Laboratories	Data collection and digital technology
	Patient pathway	Sampling criteria		
Sweden	Minimal	None	None	None
Netherlands	Modest	None	None	None
France	Modest	Modest	Modest	Modest
England	Modest	Modest	Modest	Minor
Scotland	Major	Modest	Major	Major
Portugal	Major	Major	Major	Modest
Spain	Major	Major	Major	Major

### Patient pathway

During the peak of the first pandemic wave, the number of people attending primary care decreased and parallel testing routes facilitated generalised access to testing. These conditions and greater use of telemedicine led to declines in primary care consultations. Therefore, the number of sentinel swabs collected through primary care in all countries decreased. Response to this varied across countries, including differences in balancing diagnostic testing and testing for sentinel surveillance.

In Sweden, COVID-19 testing was initially only available for healthcare workers and those with moderate or severe illness, resulting in patients with mild or no symptoms visiting general practitioners (GPs) and using the sentinel system for COVID-19 testing. Generalised access to testing, which started in June, led to major declines in participating practices.

In the Netherlands, patient pathways changed, which decreased the number of swabs taken in sentinel surveillance. GPs were organised in COVID-19 conglomerates, which reduced the number of GPs seeing symptomatic patients. Since GPs were only able to submit sentinel swabs for their own patients, the number of sentinel swabs collected decreased. Starting in June 2020, municipal health services redirected symptomatic patients from GPs to generalised testing.

In France, the number of sentinel practices did not change, although more people may have been allowed access to testing due to a change of case definition. The sentinel system ended on 18 May 2020, after the start of generalised access to COVID-19 testing.

England tripled its number of sentinel practices. Patient pathways changed, with swabs either taken by a clinician or by the patient. The sentinel system also extended its serology surveillance.

In Scotland, GPs were no longer able to carry out swabs in their usual settings, but patients were identified using a new centralised telephone triage pathway. Patients were either referred to local telephone-based COVID-19 hubs for a self-swab and home care or for a face-to-face GP assessment and swab at local COVID-19 assessment centres. As new COVID-19-specific diagnostic laboratories were set up for the general public, the system was amended to include samples collected in these parallel testing units in the enhanced surveillance system.

Portugal and Spain ended sentinel surveillance for all respiratory infections, including influenza. In Portugal, the focus shifted to diagnosis of COVID-19 cases in reference hospitals and later in COVID-19 centres supported by private laboratories. In Spain, diagnosis was extended to primary care physicians and COVID-19 testing units, and relocation of GPs to hospitals and the widespread use of telephone consultations during the most intense weeks of the pandemic led to declines in swabs taken.

#### Sampling criteria

England, Portugal, Scotland, and Spain swabbed using the WHO COVID-19 case definition [18].

France amended influenza sampling criteria from ILI syndromic surveillance to ARI.

Both Sweden and the Netherlands maintained their original sampling strategy.

All countries continued with face-to-face swabbing by physicians and in some cases practice nurses. However, only England and Scotland used self-swabbing for sentinel surveillance.

#### Laboratories

Before COVID-19, each of the countries tested sentinel swabs in centralised reference laboratories for influenza virus. England, the Netherlands and Sweden continued to test all sentinel samples at the reference laboratories.

In the Netherlands, the number of specimens collected within the sentinel surveillance system was too low to be meaningful due to generalised testing of symptomatic patients at municipal health services. The laboratory capacity was increased and decentralised for SARS-CoV-2 testing.

All the other surveyed countries shifted either during or after the peak to decentralised testing and increased laboratory capacity to test sentinel samples for SARS-CoV-2.

Initially, France continued to use three reference laboratories, but later decentralised testing outside the ‘Sentinelles network’ to be consistent with the national testing strategy.

Portugal decentralised testing and made diagnostic testing available to the general population on request from a physician.

In Scotland, testing policy changed from surveillance to diagnostic testing. All regional health boards were asked to test and process SARS-CoV-2 samples from their region through local laboratories to reduce turnaround time. In addition, new laboratories dedicated exclusively to SARS-CoV-2 testing were implemented.

In Spain, SARS-CoV-2 testing was extended to all regional laboratories with diagnostic capacity during the first pandemic phase. Some of the regional laboratories participated in the influenza reference laboratory network, but many were new participating laboratories dedicated to SARS-CoV-2 testing.

#### Data collection and digital technology

The use of digital technology for patient consultations, patient data entry and reporting of results increased across the study period.

Portugal shifted to online data collection and France made amendments to their data collection.

In France, Portugal and Scotland, centralised databases for nationwide collection of SARS-CoV-2 results were implemented.

Spain adapted the Spanish Surveillance System electronic platform (SiViES) to include surveillance for COVID-19. The former Web application of the Spanish Influenza Sentinel Surveillance System was adapted to include data from ARI sentinel surveillance in primary care.

In Portugal, the National Surveillance System (SINAVE) was used for clinical, epidemiological and laboratory data collection. In France and Scotland, information was sent directly to both the patient and the physician.

In England, results from September 2020 onwards were sent via e-Laboratories, an electronic laboratory reporting system. Data from parallel national testing sites, which were set up to allow generalised access to SARS-CoV-2 testing, were shared with GPs. These data were entered into a central database and included in sentinel surveillance.

In the Netherlands, although not sentinel based, digital technology was developed for scheduling testing appointments, collecting basic consultation data, collecting and reporting testing data and testing results of feedback to patients and GPs. The system centralised country-wide collating of all data and production of weekly reports, including sentinel GP data.

### Strengths, challenges and lessons learned

The adaptations to the sentinel surveillance systems revealed the strengths and challenges of these systems as perceived by study sites’ focal points. Moreover, in hindsight, lessons could be learned from this first pandemic phase, when changes had to be made very quickly. The strengths, challenges and lessons learned for each study site are summarised below and detailed in Supplementary Table S2. The lessons learned overlap with the perceived strengths and challenges. In addition, the lessons learned all reflect the individual perceptions of the informants.

#### Strengths

A particular strength to enable adaptation of primary care sentinel surveillance for ILI to COVID-19 was the presence of (voluntary) long-established systems and networks between GPs, laboratories and public health professionals. Flexibility and adaptability was mentioned as strength as well. Political engagement and commitment of staff for enabling efforts to maintain sentinel surveillance systems as well as having (human) resources dedicated for both programme management and clinical advice were also noted as key drivers. Other identified strengths were unique perceptions to each reporting site.

#### Challenges

The challenges that were noted predominantly included the change of patient pathways due to testing policies, resulting in lower numbers of samples taken.

In France, the decrease in samples emerged from an increase in teleconsultations.

In the Netherlands, symptomatic patients were initially tested in dedicated COVID-19 practices and later in SARS-CoV-2 testing centres.

In Sweden, diagnostic tests were initially only available through the sentinel surveillance system, which increased GP participation and sample numbers. However, availability of tests through parallel testing routes later in the pandemic led to major declines in both the number of patients attending practices and the number of participating practices.

In England, the sharp increase in GP practices resulted in challenges in terms of data quality.

Data collection was challenging in Portugal and Spain as well. For example, in Portugal, clinicians perceived data collection as time-consuming and tedious as they perceived the mandatory data collection system to be unnecessarily complicated.

Both Scotland and France faced challenges with the transportation of samples from rural and remote areas, which delayed the testing processes. In Scotland, initially multiple modes of transport were used, including couriers, motorbikes, planes and boats for rural and remote areas. Later, testing was decentralised, which sped up swab processing and sharing of results.

Shortages in PPE, swabs, reagents or extraction kits for swab processing were noted by multiple countries.

France aborted testing for other respiratory pathogens due to constraints in laboratory capacity. Lack of (dedicated) staff was mentioned as challenge as well.

#### Lessons learned

The lessons that were derived from the adaptations to primary care sentinel surveillance during this first pandemic phase could be condensed to (i) preparedness, (ii) adaptability/flexibility, (iii) improved data infrastructure and (iv) awareness of the dual purpose of testing for diagnostics and surveillance and its consequences.

Preparedness includes having or making dedicated resources available, for instance for public health teams and coordination of surveillance (Portugal, Scotland, Spain), and being prepared for high caseloads (Sweden). Flexible systems are needed to adapt quickly to new routines, such as the use of self-swabbing (England, Scotland), decentralisation of laboratory testing and the use of telephone consultations (Portugal). Scotland indicated the importance of developing and establishing new protocols and readiness of checklists to communicate to multiple stakeholders and support rapid implementation.

The importance of an effective data infrastructure was noted by Portugal, Scotland and Spain. Information systems need to be flexible, data duplication in both collecting and reporting should be avoided, and data completeness should be prioritised and fed back to teams of concern.

The dual purpose of testing resulted in new partnerships with different stakeholders, which maintained sample numbers (Scotland). Portugal noted the need to consider surveillance when making changes to patient pathways. In the Netherlands, the contribution of sentinel GP surveillance to the national testing strategy during the pandemic was considered to be too small.

## Discussion

The COVID-19 pandemic has resulted in widespread disruption to public health and healthcare systems across the world. In line with international guidelines, COVID-19 surveillance systems were set up by adapting existing respiratory sentinel surveillance systems. Here we summarise the efforts of seven primary care sentinel sites across six countries in Europe. These efforts maintained or adapted influenza sentinel surveillance to include COVID-19 during and immediately after the first pandemic phase. The extent of system adaptations were wide-ranging, varying from almost no changes to sentinel surveillance systems in Sweden and the Netherlands, to moderate changes in England and France, to the implementation of completely new systems in Scotland, and to complete cessation of sentinel surveillance in Portugal and Spain.

Many factors contributed to the drivers of change to sentinel surveillance. All the investigated countries made changes to patient pathways, which included asking the public to avoid visiting primary care settings by using digital technology and/or parallel testing routes that allowed for generalised access to diagnostic testing. These changes were made possible because of established networks and partnerships and political commitment. Countries that succeeded in maintaining or adapting sentinel surveillance throughout the first stages of the pandemic prioritised surveillance and used the data to inform decision-making.

The dual challenge arises of how to maintain COVID-19 primary care surveillance and to ensure in parallel that influenza surveillance can occur in the context of these modified respiratory patient pathways and surveillance systems. Where diagnostics are prioritised and surveillance strategies amended, questions remain about how best to restart influenza sentinel surveillance, particularly when patient pathways remain disrupted [19,20]. In Spain, efforts are ongoing to set up sentinel syndromic surveillance systems sensitive to influenza viruses, RSV and SARS-CoV-2. Changes include using ARI rather than ILI case definition in primary care, using automated data extraction from electronic health records, providing new testing pathways, using PCR tests rather than antigenic tests and re-establishing regional or establish new networks.

In the near future, SARS-CoV-2 will likely become endemic [1]. Simultaneously, however, pockets of isolated outbreaks will occur and new SARS-CoV-2 variants will emerge across the world [10]. Moreover, the hazard of an influenza epidemic perseveres [21]. Accordingly, flexibility will be required to allow countries to adapt rapidly to changing contexts and responses. Digital technology is likely to continue to play an important role in the response to COVID-19. Surveillance for COVID-19 should adapt to specific contexts and use the information gathered through generalised testing to better understand SARS-CoV-2. In addition, sentinel surveillance needs to be re-established for influenza viruses and adapted for new SARS-CoV-2 variants [21]. Therefore, generalised testing for case finding and containment should be disentangled from sentinel surveillance. This either requires retransformation to primary care testing of samples of ILI cases or to incorporate sampling for sentinel surveillance in the newly established testing routines, under the umbrella of primary care sentinel surveillance.

To foster sustainability of sentinel surveillance, the workload imposed by data collection and validation should be minimised a much as possible. Marbus et al. [22] propose that surveillance should serve both public health and patient care. More harmonisation of data standards, automatisation of data collection and validation and support of data specialists may lower the administrative burden of sentinel surveillance. Furthermore, with a new respiratory disease emerging, already at the outset of changing routes in the healthcare system, data experts should be involved to align adequate data collection. In addition, being flexible will enable upscaling from sentinel surveillance to community testing when cases surge.

A limitation of the study is that timing after the first pandemic wave and methods used for data collection did not allow for the consideration of subsequent or evolving approaches taken by countries to compensate for changes in data collection in primary care surveillance. Moreover, the rapid changes in the emergence of the virus and the policy responses in containment measures, testing processes, vaccination strategy and the like, pose substantial challenges in adapting primary care sentinel surveillance for COVID-19 while maintaining influenza surveillance. Another limitation is that the adaptations as reported in the surveys by representatives of the study sites reflect the perception of the participants. As the participants were subjected to enormous workloads, they may have overlooked the full range of changes implemented in their sentinel surveillance system. In addition, it may have been challenging to disentangle the objectives of routine surveillance from pandemic control and to provide data for research studies (e.g., vaccine effectiveness) and attributes of surveillance system functioning [22]. The subsequent classification of the extent of changes was a subjective evaluation of the researchers and did not include quantitative indicators of system functioning. Moreover, the adaptations mapped here do not represent a static and quantitative picture but rather are illustrative for the range of countries’ responses during the first half of 2020. In the year that followed, the primary care surveillance systems kept adapting to the changing situation of new outbreaks and new SARS-CoV-2 variants. These adaptations will be further subjected to evaluation in the near future.

### Conclusions

This paper takes stock of the decisions made by a selection of countries across Europe in response to the first wave of the COVID-19 pandemic. As we go forward, many of the challenges will remain. By looking at how countries have adapted primary care sentinel surveillance, we can make better and more informed decisions going forward – in particular, how best to integrate sentinel surveillance for influenza viruses and other respiratory pathogens with sentinel surveillance of SARS-CoV-2.

## Supplementary Data

217000 Supplement
