# Supplementary material for: Rapidly adapting primary care sentinel surveillance across seven countries in Europe for COVID-19 in the first half of 2020: strengths, challenges, and lessons learned
Source: Euro Surveill. 2022 Jun 30;27(26):2100864. doi: 10.2807/1560-7917.ES.2022.27.26.2100864 (PMC9248262; doi:10.2807/1560-7917.ES.2022.27.26.2100864)
Supplement: Supplement [file 21-00864_JANSEN_Supplement.pdf]

Supplement to Rapidly adapting primary care sentinel surveillance across seven countries in Europe for COIVD-19 in the first half of 2020: strengths, challenges and lessons learnt

This supplementary material is hosted by *Eurosurveillance* as supporting information alongside the article 'Rapidly adapting primary care sentinel surveillance across seven countries in Europe for COVID-19 in the first half of 2020: strengths, challenges, and lessons learned', on behalf of the authors, who remain responsible for the accuracy and appropriateness of the content. The same standards for ethics, copyright, attributions and permissions as for the article apply. Supplements are not edited by *Eurosurveillance* and the journal is not responsible for the maintenance of any links or email addresses provided therein.

Supplementary Table S1: Description of participating I-MOVE primary care COVID-19 surveillance networks

Network (country)	Participating Institutes
RCGP RSC<br>(England), SARS CoV-2 swab testing was performed in the Respiratory Virus Unit Colindale, UK the Health Security Agency (UKHSA), formerly Public Health England (PHE), and at dedicated COVID-19 community testing centres	Department of Health (DH);<br>University of Oxford (UOXF), Royal College of General Practitioners (RCGP) research and surveillance centre (RSC)
Réseau Sentinelles<br>(France)	Sorbonne Universite (SU);<br>Institut Pasteur (IP)
Irish sentinel GP network (Ireland)	Health Service Executive (HSE)
Navarra (Spain)	Organismo Autonomo Instituto de Salud Publica Y Laboral de Navarra
Nivel Primary Care Database - Sentinel Practices (Netherlands), virological testing of the samples is performed at the RIVM	Netherlands Instituut voor Onderzoek van de Gezondheidszorg (Nivel); Rijksinstituut voor Volksgezondheid en Milieu (RIVM)
Rede Médicos-Sentinela (Portugal)	Instituto Nacional de Saude dr. Ricardo Jorge (INSA)
NHS community pathways, primarily comprising COVID-19 community assessment centres and triage hubs (Scotland)	NHS National Services Scotland (NHSNS)
Spanish Sentinel Surveillance System of Acute Respiratory Infections (Spain)	Instituto de Salud Carlos III (ISCIII);
Sentinelövervakning /Sentinel Surveillance Network (Sweden)	Folkhalsomyndigheten (FOHM)

Supplementary Table S2: Changes to primary care influenza sentinel systems in response to the first phase of the COVID-19 pandemic

	Country	Description of influenza sentinel system pre-COVID-19	Changes made in response to COVID-19			
			**Participating sites; sampling criteria**	**Data collection**	**Testing process**	**Strengths/ Challenges/ Lessons**
**Minor/ no changes**	**Sweden**	- 79 primary care practices (GPs and paediatric clinics) covering 6.4% of the population.<br>- GP takes face to face swab<br>- Doctors asked to swab the first 5 ARI/ ILI patients that enter the practice per week (around 50 samples per week)<br>- Data collected by the GP during the consultation on paper and online.<br>- Data validated by the Public Health Agency of Sweden<br>- Samples transported by the public postal system.<br>- Samples tested for influenza only at the Public Health Agency of Sweden.<br>- Results shared through weekly reports	- Increase in registered sampling practices to 102 (coverage 7.9%) during the peak and reduction to 15 (coverage 1.5%) post-peak.<br>- No change in who takes the swab.<br>- No change in sampling strategy. All additional samples also tested.<br>- Influenza surveillance stopped week 15<br>- Sweden started to offer testing for everyone in June 2020 which resulted in a decline in number of swabs taken by registered GPs.	- No change	- No change	**Strengths:**<br>- Established sentinel system and protocols; reimbursement to registered GPs for first five samples.<br>- Sentinel samples were used for diagnostic purposes which encouraged attendance prior to change in patient pathway.<br>- Now using sentinel system to offer Influenza and COVID-19 testing.<br>**Challenges:**<br>- Low coverage in some counties.<br>- Change in patient pathway reduced number of samples sent through GPs.<br>- Reduction in capacity meant had to stop recruiting more GPs to register<br>- Shortage of reagents<br>**Lessons:**<br>- Be prepared for high caseload<br>- Urgency creates flexibility<br>- Difficult to obtain two swabs (diagnostic and sentinel surveillance)
	**NL**	- 40 registered ILI sentinel practices covering around 0.8% of population.<br>- GPs test the first two ILI patients on Monday through Wednesday. If no patients attend GPs test the first two	- No change in sentinel surveillance strategy or registered practices.<br>- Patient pathway changed and public was asked to stay at home if not severely unwell. GPs organised	No change	No change	**Strengths:**<br>- Established integrated epidemiological an virological sentinel system and protocols; reimbursement incentive to GPs depending on number of swabs taken.

	Country	Description of influenza sentinel system pre-COVID-19	Changes made in response to COVID-19			
			Participating sites; sampling criteria	Data collection	Testing process	Strengths/ Challenges/ Lessons
		patients with ILI or ARI from Thursday to Sunday. At least one should be under 10 yrs.<br>- Samples tested for influenza, RSV, rhinovirus, and enterovirus.<br>- Data is collected on paper, face to face and entered by the laboratory into the RIVM Laboratory Information Management System (LIMS).<br>- Samples are sent by regular mail and tested at the National Influenza Centre at the National Institute of Public Health and the Environment (RIVM).<br>- Results are entered into the LIMS by the RIVM.	collaborative consultation offices for suspect patients. This reduced the case load and number of sentinel swabs as GPs can only submit swabs of their own patients.<br>- In June post- peak testing centres have been set up further reducing the caseload.			- Sentinel swabs are used for diagnostic purposes (although can take days) which encourages patients to have them swabbed.<br>**Challenges:**<br>- Changes in patient pathway reducing consultation numbers.<br>- Testing for symptomatic patients was redirected to municipal health services, bypassing GPs.<br>- GPs formed collaboratives which reduced the case load and number of sentinel swabs as GPs can only submit swabs of own patients.<br><br>**Lessons:**<br>- Sentinel surveillance too limited to contribute to national testing strategy with rapid sample-to-result times of less than 48 hours, 7 days/week.
**Modest changes**	**France**	- 333 sentinel GPs and paediatricians (0.3% coverage) at least.<br>- GPs sample 1 ILI patient of any age and 2 elderly ARI patients. Paediatricians sample 1 ILI patient. Samples were face to face by consulting physician.<br>- Patient information collected on paper by physician during consultation. Form sent to lab and data entered onto database for secure transfer and storage to Sentinelles.	- No. of participating sites stayed same during peak. Post peak Sentinelles surveillance stopped and testing transferred over to the National Testing Strategy.<br>- During peak ILI syndromic surveillance replaced with ARI. GPs sampled 1 ARI patient <65 & 1 ARI patient 65+; paediatricians sampled 1 ARI patient.	- During peak pre-COVID-19 process followed for data collection.<br>- Post peak data collected online by participating GP and transferred using secure app or dedicated website or over phone in some cases. Sentinelles epidemiologists verified all data cross	- Public mail + other mail service to transport samples<br>- At peak NRCs tested. Post peak medical labs tested as per national testing strategy independent of Sentinelles surveillance.<br>- Post-peak labs stopped testing for some respiratory pathogens due to	**Challenges:**<br>- Disturbance in postal service<br>- Lack of PPE<br>- Increases in teleconsultations reducing number of samples<br>- Cessation of pathogen testing due to lab capacity

	Country	Description of influenza sentinel system pre-COVID-19	Changes made in response to COVID-19			
			Participating sites; sampling criteria	Data collection	Testing process	Strengths/ Challenges/ Lessons
		- Samples transported by public post.<br>- Testing at 2 national reference centres (NRC) (CNR, Paris and Lyon) and at the University of Corsica.<br>- Testing for influenza, hRV, VRS and hMPV.<br>- Results sent to swabbing physician by email and available on Sentinelles account.	- Post peak, generalised testing was made available from 18 May 2020.<br>- Post peak, sentinelles GPs were allowed to retrospectively report on SARS-CoV-2 status by modification of electronic forms by adding a question on prescription of PCR test for SARS-CoV-2.	checking with GP if needed.	capacity but continued COVID-19 and Influenza until week 13.<br>- Results sent electronically to SIDEP (centralised COVID-19 results database), swabbing physician and patient.	
	**England**	- 100 GP sentinel sites, 4-10 samples taken by the GP or practice nurse per practice per week. In addition UKHSA receives samples taken via drive through or face to face sampling through parallel testing channels set up across the UK.<br>- Sampling of patients with ILI or LRTI; except those who opt out or had recent vaccination<br>- Data collected on paper by GP practice. Some practices use automatically filled online form. Coded at practice and checked by RCGP RSC<br>- Kit sent on request from RCGP RSC liaison team who request from UKHSA) supplier.<br>- Samples tested centrally at Colindale, Respiratory Virus Unit.<br>- Test for influenza and other respiratory viruses. If negative tested further.	- During and after the peak number of sentinel sites increased to 300. 20 samples per week, more if needed. Goal, total 600 across all age bands.<br>- During the peak sampling was offered to patients presenting with COVID-19 symptoms (within 7 days of onset), recent travel. Now patients presenting with ILI or LRTI or those suspected to have or have been exposed to COVID-19 with persistent cough/ loss of taste/ smell, SOB, fever or wheezing (within ten days of onset)<br>- During the peak samples either taken face to face by GP, or self-swab (Drive through nationally provided).<br>- Also now doing serosurveillance of up to	- Processes remain the same as pre COVID-19<br>- For samples collected through the drive through or parallel face to face testing sites, patient data is screened by GP practices and included in a centralised database overseen by UKHSA.	- During the peak swabs taken in practice sent by post. Self-swabs sent by post (online voucher code piloted in 21 practices during the peak, this will be used by all from end September).<br>- Testing location unchanged.<br>- Samples only tested for COVID-19.<br>- Results sent to the GP during the peak. Going forward results sent via e-Labs system and a text to those who self-swab.<br>- Serology swabs go to the UKHSA centre in	**Strengths:**<br>- Strong relationships and network.<br>- University and RCGP support.<br>- Adaptability in primary care.<br>- Willingness to engage.<br><br>**Challenges:**<br>- Long time period from test to result.<br>- Lack of PPE. Shortages in swabs.<br>- Change in patient pathway meant patients not attending primary care.<br>- Low remuneration.<br>- No results flow for serology so patient doesn't get anything back.<br><br>**Lessons:**<br>- Importance of self-swabbing and its use.<br>- Importance of links with labs to order tests and to share results directly with patients.

	Country	Description of influenza sentinel system pre-COVID-19	Changes made in response to COVID-19			
			Participating sites; sampling criteria	Data collection	Testing process	Strengths/ Challenges/ Lessons
			1000 serology specimens per week.		Manchester and sent to test sites.	
**Major changes**	**Portugal**	- Sentinel sites (GP and A&E) across the country, some regions underrepresented. 40,000 people covered.<br>- Swabbing of all ILI patients. Around 1000 samples each season (wks 40 to 20).<br>- Face to face swabs by GP or nurses.<br>- Paper based data collection by attending physician.<br>- Validation by network coordinators with verification by GP.<br>- Samples transported by courier via the National Institute of Health (NIH).<br>- Test for influenza and other respiratory pathogens if negative.<br>- Results sent to GP or A&E focal point. Results available weekly on influenza surveillance report.	- Initially patients called national helpline for referral to reference health centre hospital (depending on clinical criteria) for swab following validation by medical Dr. Later tests at COVID-19 centres (drive through, private labs, health centres and hospitals)<br>- All suspect cases meeting ECDC case definition tested. During peak also tested high risk contacts, exposed populations.	Data entered online by medical doctor.<br>Data collected face to face or over the phone.	Initially testing centralised at National Reference Laboratory (NRL). Later SARS-CoV-2 testing was decentralised to public and private labs. Testing for Influenza and other respiratory pathogens was not performed by all testing laboratories. Dedicated transport of samples.<br>SARS-CoV-2 results notified through mandatory surveillance system.	**Strengths:**<br>- Strong GP, lab and public health network with standard protocols. High voluntary participation.<br>**Challenges:**<br>- Voluntary sentinel network made recruitment in some regions difficult.<br>- Lack of resource for coordination<br>- Medical records not available increasing burden of data collection in sentinel sites.<br>- Mandatory surveillance system not user friendly with many variables which led to reduction in data quality.<br>- Many information systems making communication challenging between levels.<br>**Lessons**:<br>- Dedicated resource for coordination<br>- If changing case management always consider surveillance. Avoid duplication of data decentralisation of telephone lines and reference centres.
	**Scotland**	- 40 sentinel influenza GP practices covering around 6% of the population<br>- Face to face swabs by the GP<br>- Up to five samples per week between weeks 40 to 20.<br>- Sampling of those with ILI at clinician discretion up to five	- Patients called NHS24 (telephone help line). If case definition met, transferred to COVID-19 Hub (CH) for telephone consultation only (if mild symptoms) or further triaged to attend COVID-19	- Paper based data collection by clinicians and some patients if self-swabbing.<br>- Data sent by email to central team for entry.	- Range of transport routes including royal mail, courier (bike, boat and air)<br>- testing decentralised to private and public	**Strengths:**<br>- Strong political buy in.<br>- Proactive communication with key front line staff.<br>- Clear protocols and readiness checklists.

	Country	Description of influenza sentinel system pre-COVID-19	Changes made in response to COVID-19			
			Participating sites; sampling criteria	Data collection	Testing process	Strengths/ Challenges/ Lessons
		per week, two under 14 yrs one 15-44 yrs and two 45 and over.<br>- Data is collected by the GP or practice nurse on paper and emailed to the Flu team.<br>- Validation by analysts on entry.<br>- Samples transported by public mail services.<br>- Testing at the West of Scotland Reference laboratory run by the NHS.<br>- Tests for Influenza and other common respiratory pathogens using multiplex PCR.	Assessment Centre (CAC) for face to face assessment (moderate symptoms).<br>- Either self swab (CH), drive through (CH or CAC) or face to face (CAC).<br>- CH and CACs asked to collect 500 swabs each, per week. Target set per health board according to population size. Initial effort to age stratify were stopped due to small sample size and change in testing policy where all had to be tested if presenting at a CAC.	- Data analysts match surveillance survey data to results and other variables.	local labs via Health Boards<br>- testing for COVID-19 only<br>- Data sent to centralised results lab system (Electronic Communication of Surveillance in Scotland (ECOSS).	- Dedicated human resource for programme management and clinical advice.<br>**Challenges:**<br>- Rapid change in patient pathway. Transportation of samples from remote rural areas.<br>- Clinician completing data surveillance form extends consultation time.<br>**Lessons:**<br>- Decentralise lab testing of samples to speed results back to patients<br>- Flexibility to adapt to political context and changing patient pathways.<br>- Prioritise data completeness feeding back to teams from the start.<br>- Importance of dedicated personnel in the national and local teams.
	**Spain**	- 772 sentinel GP (555) and paediatric practices (217) from 16 regional networks out of 19 regions covering 2.44% of the population.<br>- First two patients attending for face to face consult who meet ILI definition.<br>- Data collected by physician face to face on paper or electronic form depending on region.<br>- Validation by national and regional coordinators and GPs.	- All COVID-19 cases were reported to the National Epidemiological Surveillance Network (RENAVE).<br>- Testing strategy was changed depending on epidemiology. Initially testing of all suspect cases and then testing only of severe cases and health workers or other essential groups.<br>- Testing depends on region with some regions using	- Data collected either on paper or electronically depending on the region.<br>- Data transferred to the RENAVE through web platform (SiViES).<br>- Information collected by public health or other health care professionals, by phone or through	- Initially testing centralised at the National Influenza Reference Centre. Later decentralised to public regional laboratories.<br>- Only testing for COVID-19.<br>- Dedicated transport of samples.<br>- Mandatory notification to RENAVE.	**Strengths:**<br>- Well established system for inter-regional coordination and management of public health alerts and emergencies<br>- Strong commitment of public health staff despite workload.<br>- Flexibility to change from decentralised system to a centralised decision making system following declaration of the State of Alarm.<br>**Challenges:**<br>- Sentinel networks disrupted in all regions.

	Country	Description of influenza sentinel system pre-COVID-19	Changes made in response to COVID-19			
			Participating sites; sampling criteria	Data collection	Testing process	Strengths/ Challenges/ Lessons
		- Samples transported by courier via the regional influenza reference laboratories. A selection of samples are sent to the National Influenza Reference Center for genetic/antigenic characterisation and B lineage determination.<br>- Results sent back to physicians and regional sentinel network coordination who share nationally with National Centre of Epidemiology ISCIII via online web platform.	primary care network and others using COVID-19 testing centres.<br>- Swabs taken by GPs or clinical staff in testing centre.	face to face interviews.<br>- Validation as before.	- Data sent to centralised Spanish Surveillance Information System (SiViES)	- GPs relocated.<br>- Lack of supplies including PPE.<br>- Changes in patient pathways (telemedicine and parallel testing units) and health seeking behaviour.<br>- Heavy data reporting requirements at national level caused delays in notification and a decrease in data quality.<br>- Lack of dedicated personnel and IT resource at regional and national level.<br>**Lessons:**<br>- Importance of strong and flexible information system and IT structure.<br>- Importance of dedicated public health network and adequate HR.<br>- Avoid duplication of data reporting.

Supplement S3: Interview protocol for semi-structured interviews sentinel system adaptions

SECTION 1 – TECHNICAL DESCRIPTION OF COVID SURVEILLANCE IN FIRST WAVE

1. Can you provide a brief description of **COVID-19 sentinel surveillance**. Consider the following points:
    a. How is the system structured? Where are samples taken? What pathogens were tested? What data was collected from patients?
        i. Has this changed since initial plans were conceived? If yes, what was the change? When did this change in planning and implementation take place and why?
    b. How are sampling sites distributed across the country? How many samples are collected? What is the sampling and data collection criteria?
        i. Is this the same as the influenza sentinel surveillance sites? If not what is different and why? Has this changed over time? Is the system still running?
    c. Where do samples go for testing and how often?
        i. Is this a new system or an existing system?
    d. How are these transported to the testing sites? Is this system used routinely?
        i. Have there been any amendments?
    e. Provide a timeline for set up of the surveillance system (e.g. dates for first samples tested etc), aggregated data to allow plotting of an epicurve of the COVID-19 outbreak and key dates of changes in surveillance/testing/data collection. This would ideally use weekly numbers of tests/ positive results by week of date of sample collection. Information to allow deduplication will be requested.
    f. What factors facilitated the rapid set up of the surveillance system? What barriers were present? {Prompts if needed include on staffing, information governance, flexibility, political buy in]
    g. What has worked well using the surveillance system you have implemented? What has not worked so well?
    h. Have you evaluated the surveillance system that you put in place? If so – what lessons did you learn? If not – do you have plans to? Can you provide examples where data gathered have influenced decision making either locally or nationally? This might include data on symptoms leading to changes in case definitions?

SECTION 2 – SURVEILLANCE PLANNING FOR THE PREDICTED SECOND WAVE/FLU SEASON

2. In your own words could you describe the main differences between the Influenza and COVID-19 sentinel surveillance systems, in terms of objectives, set-up and key outputs?.

3. What are your plans for the upcoming autumn and winter season? Will you continue the same set up? If not, why not and what will be changed? What barriers or enablers are there to combining influenza and COVID-19 sentinel surveillance? What factors are driving decision making with regards to sentinel surveillance for influenza and COVID-19? [if needed as a prompt: what are the pros and cons of the different systems? What are the competing priorities between the systems? How does the sentinel systems fit within the contact tracing landscape?]

SECTION 3 – REFLECTIONS ON THE PANDEMIC SO FAR

4. Are there any additional challenges have you encountered during this pandemic in the set-up and maintenance of surveillance which we've not yet discussed?

5. What lasting lessons have you learned from the experience of the Covid that would be useful in future proofing sentinel surveillance systems going forward? Are there ways in which the system can be more flexible and able to respond quickly to a new population or pathogen?
